# Epidemiology and predictors of multidrug-resistant Enterobacteriaceae in intensive care units of a tertiary hospital in Somalia: a five-year retrospective study (2019–2024)

**DOI:** 10.3389/frabi.2026.1889216

**Published:** 2026-09-03

**Authors:** Omar Sidow Zubair, Yusuf Hared Abdi, Tigad Abdisad Ali, Zakaria Ibrahim Issack, Abdirahman Abdirizak Ahmed

**Affiliations:** 1 Department of Tropical Infectious Disease School of Postgraduate and Research, Banadir University, Mogadishu, Somalia; 2 Center for Health Research and Innovation, Somali National University, Mogadishu, Somalia; 3 Mogadishu Institute of Health, Mogadishu, Somalia; 4 Department of Public Health, Faculty of Medicine and Health Sciences, Hormuud University, Mogadishu, Somalia; 5 Department of Infection Prevention and Control, Mogadishu-Somalia-Turkey Recep Tayyip Erdoğan Training and Research Hospital, Mogadishu, Somalia; 6 SomHerd Solutions, Mogadishu, Somalia; 7 Pastoral and Agro-Pastoral Research Organization (PARO), Mogadishu, Somalia; 8 One Health Unit, Department of Health Emergencies, Ministry of Health and Human Services, Federal Government of Somalia, , Mogadishu, Somalia; 9 Centre for One Health, Somali National University, Mogadishu, Somalia

**Keywords:** antimicrobial resistance (AMR), antimicrobial stewardship, antimicrobial susceptibility test, multi drug resistance, Somalia

## Abstract

**Background:**

Multidrug-resistant Enterobacteriaceae (MDRE) represent a critical threat in intensive care units, particularly in low- and middle-income countries, where surveillance data remain scarce. This study characterized the epidemiology and predictors of multidrug resistance among Enterobacteriaceae isolates in a Somali tertiary hospital.

**Methods:**

This retrospective descriptive study analyzed 758 unique Enterobacteriaceae isolates from culture-positive specimens between January 2019 and December 2024, using disk diffusion for eight agents; For colistin, broth microdilution (CLSI M07) was applied to isolates screening non-susceptible on disk diffusion, and genera with intrinsic polymyxin resistance (*Proteus*, *Serratia*, *Morganella*) were excluded from the colistin denominator. Multidrug resistance (MDR) was defined as nonsusceptibility to ≥3 antimicrobial classes. Bivariate associations and multivariable logistic regression identified the independent predictors of MDR.

**Results:**

The overall prevalence of multidrug resistance was 37.9%. Klebsiella spp. demonstrated the highest MDR prevalence (54.9%), while Escherichia coli had lower odds of resistance (aOR, 0.27; 95% CI 0.19–0.38). Sputum specimens harbored the highest proportion of MDR isolates (44.9%), followed by blood (35.8%) and urine (19.0%). The prevalence of MDR remained relatively stable at 24–25% in 2019–2020, subsequently declined to 15% in 2021, and then escalated dramatically. Colistin retained the greatest *in vitro* activity: acquired resistance was documented in 1.2% (9/735 tested) of isolates from genera without intrinsic polymyxin resistance, all of them multidrug-resistant (9/283, 3.2%, versus 0/452 of non-MDR isolates; p = 0.001). A further 21/758 isolates (2.8%) belonged to genera intrinsically resistant to colistin. Because confirmatory broth microdilution was applied only to isolates screening non-susceptible, the colistin figure is a conservative lower-bound estimate. Tigecycline was tested in only 20.4% of isolates, among which resistance was 17.4%.

**Conclusions:**

Multidrug-resistant Enterobacteriaceae are escalating alarmingly in Somali ICUs, with ciprofloxacin resistance of 95.4% among tested MDR isolates, which severely constrains empiric therapeutic options. The urgent implementation of antimicrobial stewardship programs, restricted-use protocols for carbapenems and colistins, and strengthened infection prevention measures are essential. National engagement with the WHO GLASS surveillance is critical.

## Introduction

Antimicrobial resistance (AMR) represents a rapidly escalating global public health crisis, undermining decades of advances in infectious disease management, and threatening the return of modern medicine to the pre-antibiotic era (Adhikary et al., 2025). In 2019, the World Health Organization designated AMR as one of the top ten global health threats, acknowledging that the emergence and spread of drug-resistant pathogens jeopardized the treatment of common infections, ranging from urinary tract infections to sepsis (Cartledge et al., 2025). Among these pathogens, multidrug-resistant Enterobacteriaceae (MDRE), notably Escherichia coli, Klebsiella spp., Enterobacter spp., and Proteus spp., have garnered particular concern, especially in intensive care unit (ICU) settings where the combined pressures of broad-spectrum antibiotic use, prolonged hospitalization, and invasive procedures create an ideal environment for the selection and transmission of resistant strains (Sajid et al., 2025). The proliferation of extended-spectrum β-lactamase (ESBL)-producing and carbapenemase-producing Enterobacteriaceae compromises the efficacy of standard β-lactam and carbapenem therapies and severely restricts the therapeutic options (Sajid et al., 2025). Efflux pump overexpression and porin loss further exacerbate resistance, leaving clinicians with few alternatives and forcing reliance on older and more toxic agents (Adhikary et al., 2025).

The impact of MDRE is disproportionately severe in low- and middle-income countries (LMICs), where constrained diagnostic capacity, limited antibiotic stewardship programs, and inadequate infection-control infrastructure accelerate the spread of resistance (Siachalinga et al., 2023). Sub-Saharan Africa bears a particularly heavy burden; empirical antibiotic prescription dominates clinical practice in the absence of robust laboratory support, national surveillance frameworks are weak or nonexistent, and patients frequently present with advanced infection due to delays in care (Siachalinga et al., 2023). In this context, ICU patients face heightened risk: invasive mechanical ventilation, central venous and urinary catheterization, and repeated antibiotic exposure render them susceptible to bloodstream, urinary, and ventilator-associated respiratory infections caused by MDRE, with mortality rates significantly higher than those observed in high-income settings (Anyaegbunam et al., 2023).

Despite global initiatives, such as the WHO’s Global Antimicrobial Resistance Surveillance System (GLASS), which highlights Enterobacteriaceae as sentinel organisms for monitoring AMR trends, data remain scarce for many regions (Cartledge et al., 2025). In Somalia and the broader Horn of Africa, published evidence on ICU-level AMR epidemiology is virtually non-existent (Maduko et al., 2025). While neighboring countries, including Kenya, Ethiopia, and Sudan, have reported alarming increases in carbapenem-resistant Enterobacteriaceae, Somalia lacks coordinated national surveillance and peer-reviewed longitudinal studies (Kariuki et al., 2022). This absence of local evidence undermines clinicians’ ability to choose effective empiric therapies, hinders infection control policy development, and stalls the implementation of stewardship interventions tailored to the unique microbial ecology of Somali tertiary hospitals (Gulumbe et al., 2024).

The critical knowledge gap centers on the dearth of longitudinal data characterizing the epidemiology, temporal trends, and predictors of multidrug resistance among Enterobacteriaceae isolates from ICU patients in Somalia. Previous studies in this region have been largely cross-sectional or limited to outpatient samples, failing to capture the dynamic evolution of resistance within high-risk hospital wards over time. Understanding how resistance patterns have shifted from 2019 to 2024 and identifying factors such as specimen type, specific ICU unit, and patient demographic characteristics that predict MDRE emergence is essential for refining clinical guidelines, optimizing antibiotic stewardship strategies, and bolstering infection prevention and control measures.

To our knowledge, this five-year longitudinal dataset represents the first peer-reviewed report of ICU-level Enterobacteriaceae antimicrobial resistance trends from any healthcare facility in Somalia, and among the very few from the broader Horn of Africa region. While the descriptive epidemiological framework appropriately reflects the foundational surveillance phase in a setting with no prior local AMR data, the temporal analysis spanning 2019–2024 including the pre-pandemic, pandemic, and post-pandemic periods provides unique insights into how extraordinary disruptions in healthcare delivery interact with resistance trajectories in low-resource settings. These data constitute a baseline against which future national surveillance findings can be benchmarked, a scientific and public health value that cross-sectional or shorter-term studies cannot provide.

This study aimed to determine the epidemiology, temporal trends, and predictors of multidrug resistance among Enterobacteriaceae isolates recovered from ICU patients at Recep Tayyip Erdoğan Hospital, Somalia, over a five-year period. The specific objectives were to describe the demographic, clinical, and microbiological characteristics of these isolates, to assess the prevalence and temporal trends of multidrug resistance from 2019 to 2024, to identify independent predictors associated with multidrug resistance, and to generate robust local evidence to inform empiric therapy choices and strengthen infection-control and antimicrobial stewardship programs in Somali tertiary healthcare settings.

## Methodology

### Study design, setting, and population

This retrospective descriptive study was conducted at Recep Tayyip Erdogan Hospital, a 400-bed tertiary referral center in Mogadishu, Somalia, admitting approximately 2,000–2,500 critically ill patients annually to Emergency and General Intensive Care Units. Culture-positive isolates recorded in the laboratory information management system between January 2019 and December 2024 were systematically reviewed. Of the 4,464 initial isolates, 758 unique Enterobacteriaceae isolates representing individual patient episodes were retained after excluding duplicates (n = 2,118), non-bacterial organisms (n = 54), and non-Enterobacteriaceae Gram-negative isolates (n = 1,534). The inclusion criteria were confirmed Enterobacteriaceae identification, complete antimicrobial susceptibility profiles, and valid patient demographic and specimen source information. The extracted variables included patient age, sex, ICU type, specimen source, organism identity, and susceptibility results. Age was grouped into ≤20, 21–40, 41–60, and >60 years to permit clinically interpretable comparison across younger, early adult, middle-aged, and older patient categories. The dataset included all ICU patients captured in the laboratory information system during the study period; patients aged ≤20 years accounted for 5.0% of the cohort, but the retrospective database did not support more detailed stratification of pediatric critical care pathways. Respiratory specimens were recorded under a sputum category in the laboratory database; bronchoalveolar lavage and mini-BAL specimens were not separately represented in the extracted dataset. Only isolates with complete antimicrobial susceptibility profiles and valid demographic/specimen data were retained for analysis; records with incomplete laboratory information were excluded during data cleaning, and the final analysis therefore used a complete-case dataset. The retrospective laboratory dataset did not consistently contain admission-to-culture timing variables, and therefore isolates could not be reliably classified as community-acquired versus nosocomial using the standard 48-hour definition.

Duplicate records were removed by retaining the first recorded culture episode per patient across the full laboratory dataset, after which the cohort was restricted to Enterobacteriaceae isolates. This yields one isolate per patient and avoids over-representation of patients with repeated cultures, but has the consequence that a patient whose first recorded episode did not yield an Enterobacteriaceae isolate does not contribute a later Enterobacteriaceae isolate to the analysis.

### Bacterial isolation, identification, and antimicrobial susceptibility testing

Specimens underwent aerobic culture on blood, MacConkey, and chocolate agar at 35 °C for 18–24 hours. Preliminary identification was performed by Gram staining, oxidase testing, and conventional biochemical assays. Definitive species identification employed either matrix-assisted laser desorption/ionization time-of-flight mass spectrometry (MALDI-TOF) or extended biochemical testing, according to routine laboratory practice. Antimicrobial susceptibility testing followed Clinical Laboratory Standards Institute (CLSI) M100 performance standards applicable at the time of testing, using the Kirby-Bauer disk diffusion method on Mueller-Hinton agar (pH 7.0 ± 0.2; depth 4 ± 0.5 mm). Eight antimicrobials were tested at standardized concentrations: colistin (10 μg), meropenem (10 μg), piperacillin–tazobactam (100/10 μg), cefoperazone–sulbactam (75/75 μg), amikacin (30 μg), tigecycline (15 μg), trimethoprim–sulfamethoxazole (1.25/23.75 μg), and ciprofloxacin (5 μg). The inoculum was standardized to a 0.5 McFarland suspension (625 nm nephelometry). Inhibition zone diameters were measured and interpreted using CLSI breakpoints. Because disk diffusion is not recommended for colistin susceptibility testing in Enterobacteriaceae owing to poor agar diffusion, colistin susceptibility interpretation in this study was based on broth microdilution (BMD) using CLSI M07 reference methodology, rather than routine disk diffusion categorical interpretation.

Intrinsic resistance phenotypes were applied in accordance with the CLSI M100 intrinsic-resistance tables prior to analysis. *Proteus* spp. (n = 15), *Serratia* spp. (n = 5) and *Morganella morganii* (n = 1) are intrinsically resistant to polymyxins as a consequence of constitutive lipid A modification with 4-amino-4-deoxy-L-arabinose, encoded by the *arnBCADTEF* (*pmrHFIJKLM*) operon, which neutralizes the anionic charge of the lipopolysaccharide and prevents polymyxin binding. These 21 isolates (2.8% of the cohort) were categorized as colistin-resistant on intrinsic grounds and excluded from the denominator used to estimate acquired colistin resistance (corrected denominator n = 737), irrespective of the minimum inhibitory concentration obtained *in vitro*. Colistin results were interpreted using CLSI M100 criteria for *Enterobacterales*, under which only intermediate (MIC ≤2 µg/mL) and resistant (MIC ≥4 µg/mL) categories are recognized and no susceptible category is defined; colistin non-susceptibility therefore denotes MIC ≥4 µg/mL. During data cleaning for the present revision, an extraction error was identified whereby isolates of intrinsically resistant genera had been carried into the colistin denominator; all colistin analyses, tables and figures have been recomputed following correction.

Not every agent was tested against every isolate in routine practice. Testing coverage ranged from 99.7% for colistin and 95.0% for meropenem to 20.4% for tigecycline and 15.0% for trimethoprim–sulfamethoxazole. All susceptibility proportions reported here therefore use the number of isolates actually tested for that agent as the denominator, and this denominator is stated alongside every estimate; isolates not tested were excluded from the corresponding calculation rather than treated as non-susceptible. Where coverage was low, testing was clinically directed rather than systematic — agents such as tigecycline were tested preferentially in isolates already known to be extensively resistant and the resulting resistance proportions are likely to overestimate resistance in the cohort as a whole.

The retrospective database did not consistently record sputum specimen-quality indicators, and sputum quality could not be formally evaluated in this analysis. Endotracheal aspirates and sterile body fluid specimens were not available as separately analyzable categories in the extracted dataset and therefore were not reported independently in the present analysis.

### Quality assurance

Multidrug resistance was defined as non-susceptibility to at least one agent in three or more antimicrobial classes and was assigned in the laboratory information system from the full routine susceptibility panel reported for each isolate. Eight agents representing eight distinct classes colistin, meropenem, piperacillin–tazobactam, cefoperazone–sulbactam, amikacin, tigecycline, trimethoprim–sulfamethoxazole and ciprofloxacin were extracted for the present analysis. MDR status as reported here therefore reflects the complete panel available to the reporting laboratory and is not derived solely from these eight agents.

### Data analysis

Descriptive statistics were computed using Stata 17.0 (StataCorp, College Station, TX). Categorical variables were summarized using frequencies and percentages, and continuous variables were presented as medians with interquartile ranges (IQR). Bivariate associations between multidrug resistance status and categorical predictors (age group, sex, ICU type, specimen source, and organism identity) were assessed using Pearson’s χ² test, with unadjusted odds ratios (OR) and 95% confidence intervals (CI) calculated for variables with p < 0.20. Multivariable logistic regression using the forced-entry method estimated adjusted odds ratios (AOR) for MDR predictors, with multicollinearity assessed via variance inflation factors (VIF > 5) and model fit evaluated using the Hosmer–Lemeshow test (p > 0.05 indicating acceptable fit). All inferential statistical analyses were performed using Stata 17.0, with significance set at p < 0.05. Temporal trend visualization and resistance pattern graphics were generated in R (version 4.3.2, R Core Team, Vienna, Austria) using the ggplot2 package. For species-level multivariable analyses, *Pantoea* spp. (n = 2), *Citrobacter* spp. (n = 4) and *Serratia* spp. (n = 5) were retained in the model, but their individual odds ratio estimates should be interpreted with caution owing to small cell sizes and wide confidence intervals. The single *Morganella morganii* isolate was non-MDR and was dropped automatically from the multivariable model because of perfect prediction, giving an analytical sample of n = 757 for **Table 1** compared with n = 758 for the descriptive and bivariate analyses. The primary inferences of the multivariable analysis are drawn from Klebsiella spp., E. coli, Proteus spp., and Enterobacter spp., which together account for the vast majority of isolates. Important clinical risk factors for multidrug resistance, including prior antibiotic exposure, comorbidities, invasive device use, length of hospital stay, mechanical ventilation, dialysis requirement, and previous healthcare contact, were not available in the retrospective laboratory database and could not be included in the regression model.

**Table 1:** Multivariable logistic regression of factors associated with multidrug resistance (MDR) among Enterobacteriaceae isolates (n = 757).

Predictor	Category (Ref.)	AOR (95% CI)	p-value
Sex	Male (ref: Female)	1.08 (0.77–1.51)	0.668
Age group (years)	21–40 (ref: ≤20)	1.05 (0.50–2.24)	0.892
	41–60	1.24 (0.57–2.70)	0.590
	>60	0.92 (0.43–1.98)	0.838
ICU type	General ICU (ref: Emergency ICU)	1.13 (0.82–1.56)	0.453
Sample type	Sputum (ref: Blood)	1.40 (0.90–2.17)	0.136
	Urine (ref: Blood)	0.61 (0.34–1.09)	0.097
Organism type	Proteus (ref: Klebsiella)	0.19 (0.05–0.70)	0.012*
	E. coli (ref: Klebsiella)	0.27 (0.19–0.38)	<0.001*
	Serratia (ref: Klebsiella)	0.22 (0.02–1.99)	0.176
	Enterobacter (ref: Klebsiella)	1.02 (0.38–2.79)	0.962
	Citrobacter (ref: Klebsiella)	0.24 (0.02–2.41)	0.228
	Pantoea (ref: Klebsiella)	1.00 (0.06–16.59)	0.999
Model statistics			
	LR χ²(13) = 105.49, p < 0.001		
	Pseudo R² = 0.105		

Groups with fewer than 10 isolates (Serratia spp., Citrobacter spp., Pantoea spp.) are shown in italics; their estimates are statistically unstable and are reported for completeness only. The single Morganella morganii isolate was non-MDR and was dropped automatically from the model because of perfect prediction, giving an analytical sample of n = 757 against n = 758 for the descriptive and bivariate analyses.

The asterisk (*) denotes statistical significance at <0.05, p<0.05.

Annual multidrug resistance proportions were accompanied by Wilson 95% binomial confidence intervals, and the linear trend across calendar years was assessed using the Cochran–Armitage test for trend.

### Ethics approval and consent to participate

Ethical approval for this study was obtained from the Research Ethics Committee of Mogadishu Somalia-Turkey Recep Tayyip Erdoğan Training and Research Hospital, Mogadishu, Somalia (Decision No. 1184; approval date: February 16, 2025). The committee waived the requirements for informed consent and consent to participate because the study involved retrospective analysis of routinely collected fully de-identified microbiological records without direct patient contact or intervention. All data were handled confidentially with restricted access, and no personally identifiable information was disclosed at any stage of the study. The study was conducted in accordance with the Declaration of Helsinki (2013 revision) and applicable international ethical standards for human research.

## Results

**Figure 1:**
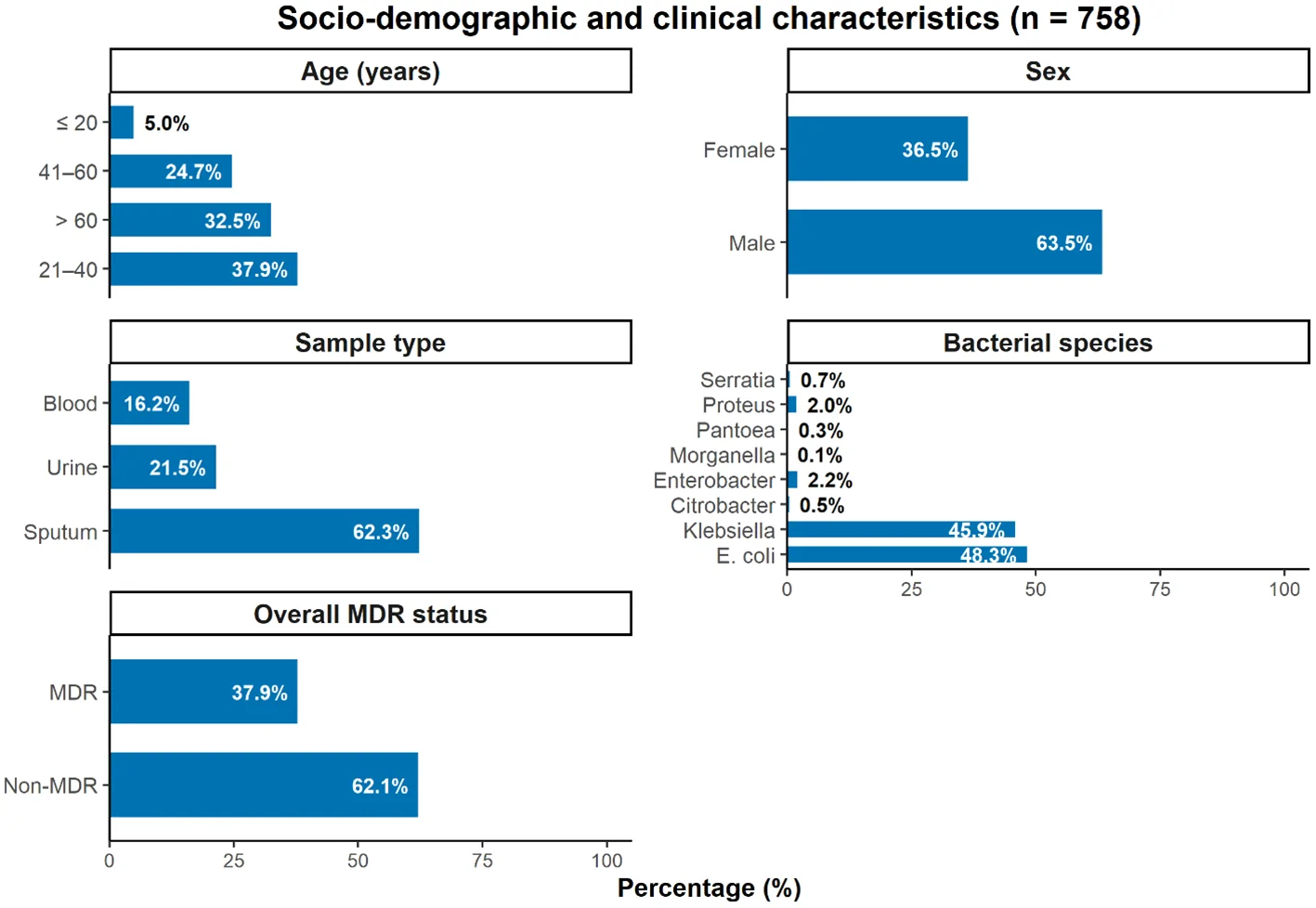
Distribution of socio-demographic and clinical characteristics among study participants.

The sociodemographic and clinical characteristics of the 758 Enterobacteriaceae isolates included in this study are summarized in **Figure 1**. The majority of isolates were obtained from male patients (63.5%), whereas females accounted for 36.5% of the study population. Age was grouped into ≤20, 21--40, 41--60, and >60 years to permit clinically interpretable comparison across younger, early adult, middle-aged, and older patient categories. The age distribution showed that patients aged 20 years or younger constituted 5.0% of the cohort, those aged 21–40 years accounted for 37.9%, those aged 41–60 years accounted for 24.7%, and those aged over 60 years accounted for 32.5%. Sample type analysis demonstrated that the majority of isolates were recovered from sputum specimens (62.3%), followed by urine (21.5%), and blood (16.2%), reflecting the predominance of respiratory tract infections among critically ill patients in the ICU setting. In terms of bacterial species distribution, Escherichia coli and Klebsiella were the most frequently isolated pathogens, representing 48.3% and 45.9% of all isolates, respectively. Other Enterobacteriaceae species, including Enterobacter (2.2%), Proteus (2.0%), Serratia (0.7%), Citrobacter (0.5%), Pantoea (0.3%), and Morganella (0.1%) were isolated at considerably lower frequencies. The overall prevalence of multidrug resistance (MDR) among the isolates was 37.9%, indicating that more than one-third of Enterobacteriaceae infections in the ICU exhibited resistance to multiple antimicrobial agents, whereas 62.1% remained non-MDR. These findings underscore the substantial burden of MDR Enterobacteriaceae in this resource-limited tertiary care setting, and highlight the need for enhanced infection control measures and antimicrobial stewardship interventions.

**Figure 2:**
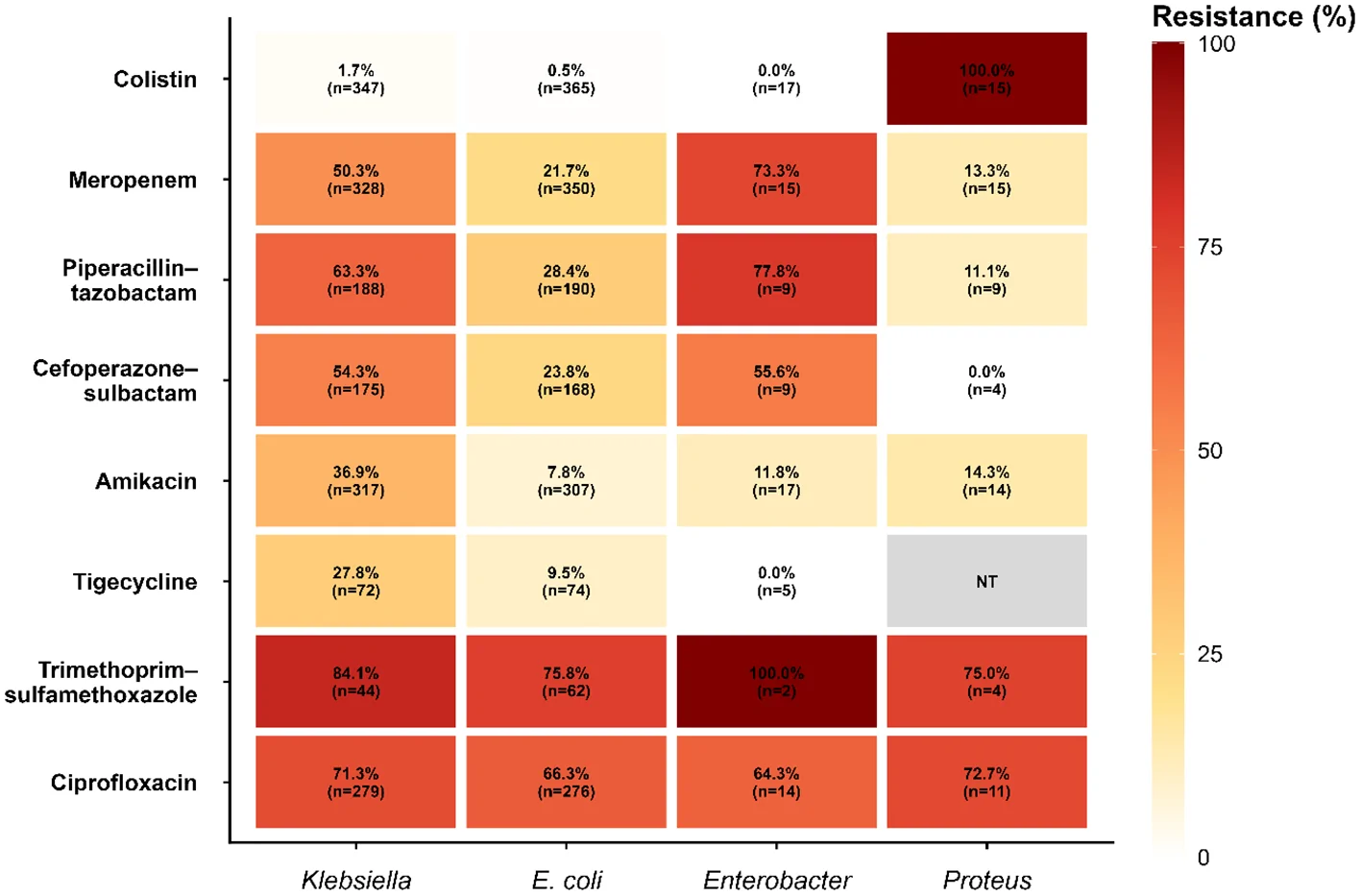
Percentage of bacterial isolates resistant to selected antibiotics by organism.

**Figure 2**. Percentage of isolates resistant to each tested antimicrobial, by organism. Denominators are the isolates actually tested for each organism–agent pair and are shown above each bar; total isolates were *Klebsiella* spp. n = 348, *E. coli* n = 366, *Enterobacter* spp. n = 17 and *Proteus* spp. n = 15. Hatched bars denote intrinsic resistance: *Proteus* spp., *Serratia* spp. and *Morganella morganii* are intrinsically resistant to polymyxins and are displayed as 100% colistin-resistant; these isolates were excluded from pooled colistin estimates. Estimates based on fewer than 10 tested isolates are shown in grey and should not be interpreted. Colistin was interpreted by broth microdilution (CLSI M07); under CLSI M100 no susceptible category exists for colistin against *Enterobacterales*.

**Figure 2** shows the proportion of isolates resistant to each of the eight tested agents, by organism, with the number tested given for each organism–agent pair. Resistance to ciprofloxacin was high across all four principal genera, at 71.3% in *Klebsiella* spp. (n = 279 tested), 66.3% in *E. coli* (n = 276), 72.7% in *Proteus* spp. (n = 11) and 64.3% in *Enterobacter* spp. (n = 14). Trimethoprim–sulfamethoxazole resistance was likewise high, at 84.1% in *Klebsiella* spp. and 75.8% in *E. coli*, although this agent was tested in only 15.0% of isolates and these estimates are correspondingly imprecise. Meropenem resistance was highest in *Enterobacter* spp. (73.3%, n = 15) and *Klebsiella* spp. (50.3%, n = 328), and lowest in *Proteus* spp. (13.3%, n = 15) and *E. coli* (21.7%, n = 350). Piperacillin–tazobactam resistance ranged from 11.1% in *Proteus* spp. to 77.8% in *Enterobacter* spp., and amikacin resistance from 7.8% in *E. coli* to 36.9% in *Klebsiella* spp. Colistin retained the greatest activity, with resistance of 1.7% in *Klebsiella* spp. (n = 347), 0.5% in *E. coli* (n = 365) and none in *Enterobacter* spp. (n = 17). *Proteus* spp., *Serratia* spp. and *Morganella morganii* are intrinsically resistant to polymyxins and are shown as uniformly resistant in **Figure 2**; their colistin results are not interpretable as acquired resistance and are excluded from all pooled colistin estimates.

**Table 2** summarizes the bivariate relationships between multidrug resistance (MDR) status and key patient, specimen, and organism characteristics for the 758 isolates included in this study. There were no statistically significant differences in the prevalence of MDR according to sex (35.4% in females vs. 39.3% in males; χ²=1.14, p=0.285), age group (p=0.898), or ICU type (35.8% in the emergency ICU vs. 40.3% in the general ICU; χ²=1.59, p=0.208). In contrast, the distribution of MDR varied markedly according to sample type (χ²=34.81, p<0.001). Sputum specimens had the highest proportion of MDR isolates (44.9%), followed by blood samples (35.8%), whereas urine samples had the lowest proportion (19.0%).

**Table 2:** Bivariate associations between multidrug resistance (MDR) status and key predictors (n = 758).

Variable	Category	Non-MDR n (%)	MDR n (%)	χ² (df)	p-value
Sex	Female	179 (64.6)	98 (35.4)	1.14 (1)	0.285
	Male	292 (60.7)	189 (39.3)		
Age group (years)	≤ 20	24 (63.2)	14 (36.8)	0.59 (3)	0.898
	21–40	174 (60.6)	113 (39.4)		
	41–60	116 (62.0)	71 (38.0)		
	> 60	157 (63.8)	89 (36.2)		
ICU type	Emergency ICU	265 (64.2)	148 (35.8)	1.59 (1)	0.208
	General ICU	206 (59.7)	139 (40.3)		
Sample type	Blood	79 (64.2)	44 (35.8)	34.81 (2)	< 0.001*
	Sputum	260 (55.1)	212 (44.9)		
	Urine	132 (81.0)	31 (19.0)		
Organism	Klebsiella	157 (45.1)	191 (54.9)	86.73 (7)	< 0.001*
	Proteus	12 (80.0)	3 (20.0)		
	E. coli	285 (77.9)	81 (22.1)		
	Serratia	4 (80.0)	1 (20.0)		
	Enterobacter	8 (47.1)	9 (52.9)		
	Citrobacter	3 (75.0)	1 (25.0)		
	Morganella	1 (100)	0 (0.0)		
	Pantoea	1 (50.0)	1 (50.0)		

Pearson’s chi-square test was used. Significant associations (p < 0.05) were observed for sample type and organism type only.

The bold values indicate statistically significant p<0.05. Statistically significant associations were observed for sample type and organism type only. The asterisk (*) denotes statistical significance at <0.05, p<0.05.

Organism identity was also strongly associated with MDR status (χ²=86.73, p<0.001). Over half of the Klebsiella isolates were multidrug resistant (54.9%), as was the majority of Enterobacter isolates (52.9%). In comparison, Escherichia coli and other less common genera (Proteus, Serratia, Citrobacter, Morganella, and Pantoea) showed substantially lower MDR rates, ranging from 0% (in Morganella) to 25% (in Citrobacter), with E. coli at 22.1%. These findings indicate that both the type of clinical specimen and the bacterial species are important predictors of multidrug resistance in this ICU setting.

Annual denominators were n = 78 (2019), 101 (2020), 70 (2021), 176 (2022), 180 (2023) and 153 (2024); total n = 758. Error bars represent 95% Wilson binomial confidence intervals. Full annual counts are given in **Supplementary Table 1**.

**Figure 3:**
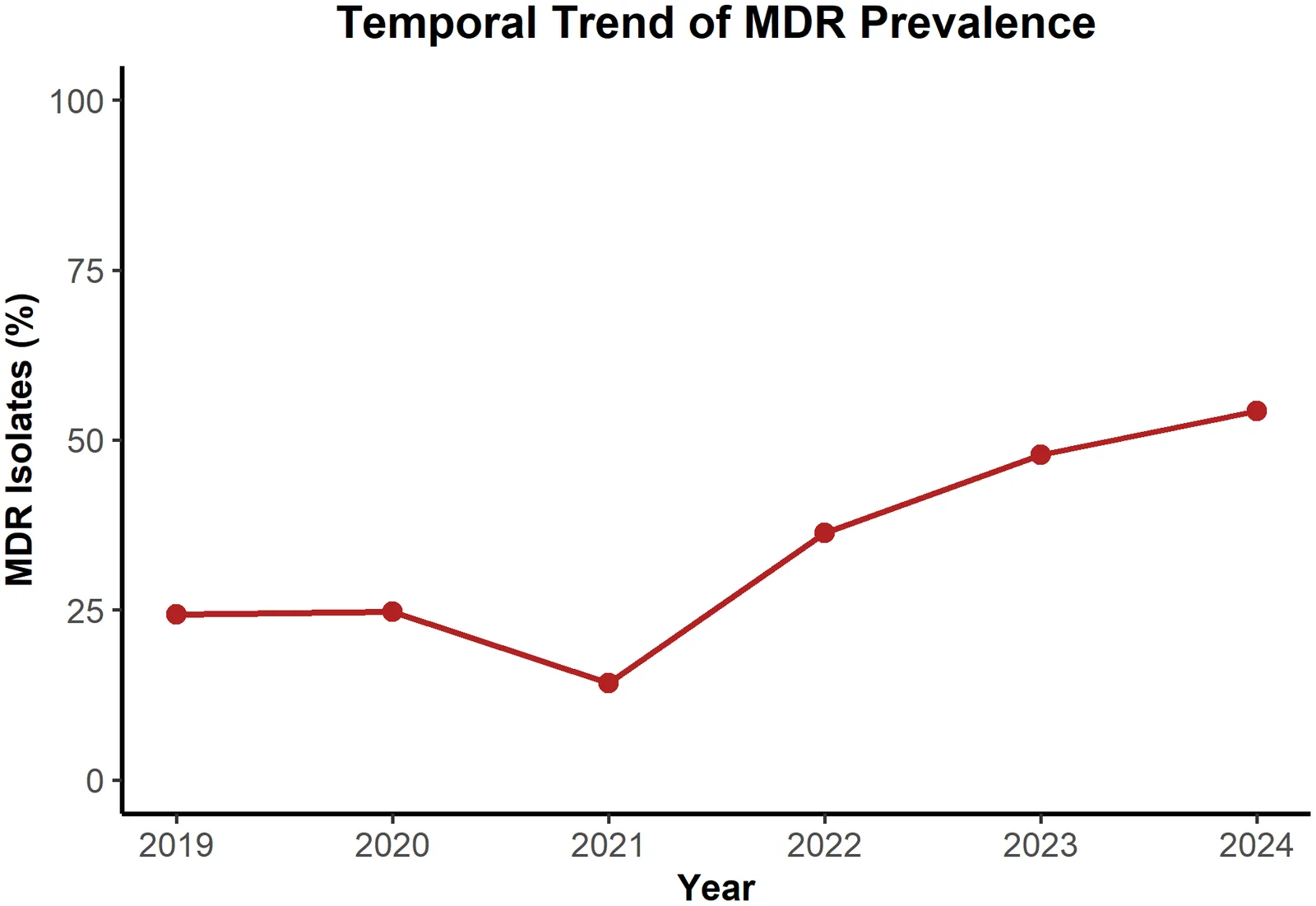
Annual prevalence of multidrug resistance among Enterobacteriaceae isolates from ICU patients, 2019–2024.

Annual denominators ranged from 70 isolates in 2021 to 180 in 2023 (**Supplementary Table 1**), and the corresponding Wilson 95% confidence intervals are shown in **Figure 3**. The intervals for 2021 (7.9–24.3%) and 2019 (16.2–34.9%) are appreciably wider than those for later years, reflecting smaller annual sample sizes, and the apparent 2021 nadir should therefore be interpreted with corresponding caution. The increase across the study period was statistically significant on a test for linear trend (Cochran–Armitage χ² = 43.91, df = 1, p < 0.001), and the 2024 estimate (54.2%, 95% CI 46.3–61.9) does not overlap the 2021 estimate.

Before presenting the multivariable results, an important interpretive caveat should be noted. Four organism groups contributed very small numbers of isolates: *Morganella morganii* (n = 1), *Pantoea* spp. (n = 2), *Citrobacter* spp. (n = 4) and *Serratia* spp. (n = 5), together representing 12 of 758 isolates (1.6%). Estimates for these groups are statistically unstable, their confidence intervals span two or more orders of magnitude, and they carry effectively no statistical power to detect or exclude an association with multidrug resistance. They are retained in **Table 1** solely for completeness and transparency of reporting; no inference of any kind should be drawn from their point estimates. All substantive conclusions of the multivariable analysis rest on *Klebsiella* spp. (n = 348), *E. coli* (n = 366), *Enterobacter* spp. (n = 17) and *Proteus* spp. (n = 15), which together account for 746 of 758 isolates (98.4%). A similar caution applies to *Enterobacter* spp. and *Proteus* spp., whose confidence intervals, while excluding unity in the case of *Proteus*, remain wide.

**Table 1** presents the adjusted odds ratios (AORs) from a multivariable logistic regression model to evaluate the predictors of multidrug resistance (MDR) among 757 Enterobacteriaceae isolates. Neither patient sex nor age group was significantly associated with MDR, with AORs close to unity for males versus females (AOR, 1.08; 95% CI 0.77–1.51; p=0.668), and for all older age categories compared to ≤20 years (p>0.5 for each). ICU type also showed no significant effect (AOR 1.13; 95% CI 0.82–1.56; p=0.453). Regarding specimen type, sputum isolates had a non-significant trend toward higher odds of MDR than blood (AOR, 1.40; 95% CI, 0.90–2.17; p=0.136), whereas urine isolates tended toward lower odds (AOR, 0.61; 95% CI 0.34–1.09; p=0.097). In contrast, organism identity emerged as a strong independent predictor. Relative to Klebsiella spp., Proteus isolates exhibited significantly lower odds of MDR (AOR 0.19; 95% CI 0.05–0.70; p=0.012), and Escherichia coli isolates had markedly reduced odds (AOR 0.27; 95% CI 0.19–0.38; p<0.001). The overall model was statistically significant (LR χ²(13)=105.49; p<0.001) and explained approximately 10.5% of the variance in MDR status (pseudo R²=0.105), indicating that bacterial species are the primary drivers of multidrug resistance in this cohort.

As noted above, the confidence intervals for *Serratia* spp. (0.02–1.99), *Citrobacter* spp. (0.02–2.41) and *Pantoea* spp. (0.06–16.59) are all compatible with both strong protection and strong risk, and support no species-level inference.

**Table 3** compares resistance between MDR and non-MDR isolates for the eight agents tested, using as the denominator the isolates actually tested for each agent. Colistin retained the greatest activity: among the 737 isolates from genera without intrinsic polymyxin resistance, resistance was absent in non-MDR isolates (0/452) and present in 3.2% of MDR isolates (9/283; χ² = 12.04, p = 0.0005). All nine colistin-resistant isolates were multidrug-resistant, comprising six *Klebsiella* spp., two *E. coli* and one *Pantoea* sp. Meropenem resistance rose from 9.5% among non-MDR isolates to 77.5% among MDR isolates, amikacin from 1.9% to 54.8%, piperacillin–tazobactam from 19.4% to 94.3%, and cefoperazone–sulbactam from 11.3% to 83.9% (all p < 0.001). Ciprofloxacin resistance reached 95.4% among tested MDR isolates and trimethoprim–sulfamethoxazole resistance 92.9%, leaving virtually no oral or first-line therapeutic option for this group. Tigecycline resistance was 36.7% among tested MDR isolates, but the agent was tested in only 20.4% of the cohort and this estimate should be regarded as provisional. Consistent with CLSI M100, isolates with colistin MIC ≤2 µg/mL are reported as intermediate rather than susceptible. All 21 isolates of *Proteus* spp., *Serratia* spp. and *Morganella morganii* were classified as colistin-resistant on intrinsic grounds and are reported separately.

**Table 3:** Antimicrobial resistance by multidrug resistance (MDR) status among Enterobacteriaceae isolates.

Antimicrobial	Coverage (% tested)	Non-MDR tested	Non-MDR resistant, n (%)	MDR tested	MDR resistant, n (%)	χ² (df = 1)	p
Colistin	99.7	452	0 (0.0)	283	9 (3.2)	12.04	0.0005
Meropenem	95.0	444	42 (9.5)	276	214 (77.5)	341.28	<0.001
Piperacillin–tazobactam	53.2	263	51 (19.4)	140	132 (94.3)	203.72	<0.001
Cefoperazone–sulbactam	47.2	221	25 (11.3)	137	115 (83.9)	184.30	<0.001
Amikacin	88.0	415	8 (1.9)	252	138 (54.8)	252.90	<0.001
Tigecycline	20.4	106	9 (8.5)	49	18 (36.7)	16.67	<0.001
Trimethoprim–sulfamethoxazole	15.0	72	50 (69.4)	42	39 (92.9)	7.18	0.007
Ciprofloxacin	77.8	350	175 (50.0)	240	229 (95.4)	133.94	<0.001

## Discussion

### Overview of key findings

This study characterized the epidemiology and temporal trends of multidrug-resistant Enterobacteriaceae (MDRE) among 758 unique isolates recovered from intensive care unit patients at a tertiary hospital in Somalia over a five-year period (2019–2024). The overall prevalence of multidrug resistance was 37.9%, indicating that more than one-third of Enterobacteriaceae infections demonstrated resistance to multiple antimicrobial agents, a finding consistent with the alarming trends documented across sub-Saharan Africa and highlighting the critical burden of drug-resistant pathogens in resource-limited ICU settings. Notably, a marked U-shaped temporal trajectory emerged, with the MDR prevalence remained stable at 24–25% during 2019–2020 (24.4% and 24.8%), declined to 14.3% in 2021, then rose to 36.4% (2022), 47.8% (2023) and 54.2% (2024). This resurgence indicates a deteriorating resistance landscape; the drivers of both the earlier decline and the subsequent increase could not be determined from the available data. In the multivariable analysis, bacterial species identity emerged as the primary independent predictor of multidrug resistance; Klebsiella spp. isolates exhibited the highest resistance burden (54.9%), whereas Escherichia coli demonstrated markedly lower odds of MDR (adjusted odds ratio, 0.27; 95% confidence interval 0.19–0.38; p<0.001). Furthermore, specimen type showed a significant bivariate association with sputum specimens harboring the highest proportion of MDR isolates (44.9%) compared with blood and urine samples. These findings provide robust local evidence that Klebsiella-driven resistance and respiratory tract infections represent the most pressing threats to effective empiric therapy in this ICU, necessitating the urgent implementation of targeted antimicrobial stewardship interventions and infection prevention measures.

The predominance of sputum-category specimens likely reflects the high burden of suspected lower respiratory tract infection among critically ill ICU patients in this setting.

### Comparison with regional and global evidence

#### MDR prevalence and species patterns

The overall multidrug resistance prevalence of 37.9% documented in this Somali ICU study is broadly consistent with, though lower than, several reported rates from other sub-Saharan African settings, yet remains within the range observed across the region (Bitew and Tsige, 2020; Tekele et al., 2021; Alemayehu et al., 2023). In Ethiopia, systematic reviews and meta-analyses have documented that MDRE prevalence rates vary considerably by setting and specimen type; a 2023 systematic review reported that among Enterobacteriaceae isolates from human sources, approximately 68% of Klebsiella spp. demonstrated multidrug resistance, with 25% to 89% of Escherichia coli isolates resistant to multiple antimicrobial agents across Ethiopian facilities (Tweldemedhin et al., 2022; Abayneh et al., 2023). A cross-sectional study in Addis Ababa identified 68.6% of Enterobacteriaceae as multidrug-resistant, substantially higher than the current Somalia-derived figure, possibly reflecting differences in ICU case-mix severity, patient demographics, or healthcare facility infrastructure (Tekele et al., 2021). In Kenya, a retrospective analysis from a Nairobi hospital revealed exceptionally high rates of resistance to penicillin/β-lactamase inhibitor combinations (91.2%) among Enterobacteriaceae isolates, mirroring the resistance patterns documented in this study (Lord et al., 2021). The World Health Organization’s Global Antimicrobial Resistance and Use Surveillance System (GLASS) framework has enabled comparison of resistance trends across participating African nations; a 2023 systematic review examining GLASS implementation across Africa noted that while robust surveillance data remain limited in many sub-Saharan African countries, available datasets confirm that *Klebsiella* spp. consistently rank among the most resistant Gram-negative pathogens (Hope et al., 2024). The dominance of Klebsiella pneumoniae as the species exhibiting the highest resistance burden (54.9%) in this study is particularly notable and represents a critical distinction from reports in some high-income settings where Escherichia coli resistance remains prominent (Tweldemedhin et al., 2022; Abayneh et al., 2023). This species-specific pattern aligns with Ethiopian findings, wherein Klebsiella spp. demonstrated higher multidrug resistance than E. coli across multiple sources, suggesting that regional ecological, epidemiological, or antimicrobial stewardship factors may preferentially select for Klebsiella resistance in East African ICU environments. The substantially lower odds of multidrug resistance observed in E. coli (adjusted odds ratio 0.27; 95% confidence interval 0.19–0.38) mirrors the findings from Kenya and Ethiopia, wherein E. coli generally exhibited lower proportional resistance compared to Klebsiella spp., despite the former’s global prominence as a source of resistant infections (Bitew and Tsige, 2020).

### Specimen and infection site trends

The predominance of sputum specimens in this cohort (62.3%) is indicative of ventilator-associated or nosocomial lower respiratory tract infections as the principal source of bacterial isolates, which is consistent with the epidemiological literature documenting a high incidence of ventilator-associated pneumonia (VAP) and ventilator-associated lower respiratory tract infections (VA-LRTI) in ICU populations (Martin-Loeches, 2020; Reyes et al., 2023). Mechanically ventilated patients represent a uniquely high-risk population for respiratory tract colonization with multidrug-resistant organisms, owing to impaired host defenses, prolonged hospitalization, and frequent exposure to broad-spectrum antimicrobials (Martin-Loeches, 2020). The higher proportion of multidrug resistance documented in sputum specimens (44.9%) than in blood and urine samples reflects the epidemiological reality that respiratory tract infections in ICU settings frequently involve highly resistant pathogens, particularly gram-negative bacilli such as Klebsiella, Pseudomonas, and Acinetobacter species (Bitew and Tsige, 2020). During the COVID-19 pandemic, elevated incidence of bacterial VA-LRTI was documented across multiple international cohorts; a multicenter European study reported VA-LRTI incidence of 50.5% among mechanically ventilated SARS-CoV-2 patients, with Gram-negative bacilli including Klebsiella pneumoniae (19.1% of isolated bacteria) and Pseudomonas aeruginosa (21.2%) identified as predominant pathogens (Bitew and Tsige, 2020; Hedberg et al., 2022; Reyes et al., 2023). In contrast, studies from settings where urinary tract infections dominate the clinical specimen distribution reflect differing ICU patient populations, procedural intensities, and infection patterns, and the Nairobi hospital study incorporating specimens across multiple anatomical sites demonstrated that while Enterobacteriaceae resistance was widespread, specimen type influenced the prevalence of particular species and resistance mechanisms (Bitew and Tsige, 2020; Lord et al., 2021). Thus, the Somali ICU specimen composition reflects typical high-intensity critical care environments characterized by prolonged mechanical ventilation and nosocomial acquisition of multidrug-resistant pathogens.

### Temporal trends (2019–2024) and post-pandemic context

The temporal trajectory documented in this study, with the prevalence of MDR declining from 24 to 25% in 2019–2020 to 15% in 2021, followed by a dramatic escalation to 54% by 2024, provides crucial insights into the complex interplay between stewardship interventions, pandemic-related pressures, and evolving resistance landscapes. The initial decline observed in 2021 rests on the smallest annual sample in the series (n = 70; 95% CI 7.9–24.3%) and is temporally compatible with several explanations that the present dataset cannot distinguish, including transient benefits of intensified infection prevention measures during the acute phase of the COVID-19 pandemic, reduced patient volume and inter-hospital transfer, and altered specimen submission practice during a period of constrained laboratory activity. Notably, the specimen mix in 2021 differed markedly from surrounding years, with sputum accounting for 20 of 70 isolates compared with 127 of 176 in 2022; since sputum isolates carried the highest multidrug resistance prevalence in this cohort, part of the apparent 2021 decline may reflect this compositional shift rather than a change in underlying resistance. However, the subsequent resurgence to 54% resistance by 2024 is consistent with global post-pandemic AMR trends, and aligns with documented disruptions in stewardship programs, increased antimicrobial consumption, and heightened selection pressure for resistant organisms in the aftermath of the pandemic. A 2024 analysis from the WHO GLASS surveillance system demonstrated statistically significant positive correlations between beta-lactam/cephalosporin and fluoroquinolone consumption and increased recovery of resistant E. coli and Klebsiella spp. from bloodstream infections across participating countries; for every one-unit increase in the defined daily dose of cephalosporin consumption, recovery of cephalosporin-resistant E. coli and Klebsiella spp. increased by 11–22% (Ajulo and Awosile, 2024; Hope et al., 2024). This pharmacoepidemiological relationship offers one plausible framework within which the escalation observed after 2021 might be understood, but antimicrobial consumption data were not available for this institution and the applicability of that relationship to the present cohort was not tested. The proposition that broad-spectrum antimicrobial use intensified locally during this period is a hypothesis suggested by the temporal pattern rather than a finding of this study. Ethiopian sepsis surveillance data collected between October 2019 and September 2020 across four referral hospitals revealed multidrug resistance frequencies of 64–72% among Enterobacteriaceae and documented continued high resistance rates during the pandemic period, suggesting that sub-Saharan African ICUs faced sustained or worsening resistance challenges rather than temporary improvements (Legese et al., 2022). The resurgence to 54% resistance in Somalia by 2024 must be contextualized within regional implementation gaps in antimicrobial stewardship; according to the World Health Organization’s Tracking AMR Country Self-Assessment Survey conducted in 2021, only 32% of African countries had established national systems for monitoring antimicrobial use, and only 58% reported having functional infection prevention and control programs at select health facilities (Gahimbare et al., 2024). These structural deficits, combined with resource constraints characteristic of low- and middle-income settings in sub-Saharan Africa, suggest that the initial stewardship gains if they occurred in Somalia were not sustainable without robust institutional infrastructure and continuous quality improvement mechanisms.

The interpretation offered above should be regarded as hypothesis-generating rather than explanatory. This study documented a temporal association between calendar period and multidrug resistance prevalence, which was statistically robust on a test for trend (χ² = 43.91, p < 0.001); it did not, however, measure any of the mechanisms proposed to underlie that association. Hospital operational indicators annual ICU admissions, bed occupancy, nurse- and physician-to-patient ratios, antimicrobial consumption in defined daily doses, hand-hygiene compliance and infection prevention program activity were not recorded in the retrospective laboratory dataset, and none of the pathways invoked here could therefore be quantified, tested or ranked. Alternative explanations consistent with the same data cannot be excluded, including changes in ICU case-mix and referral patterns over the six-year period, shifts in specimen submission or culture-request practice, changes in laboratory throughput and capacity, and secular trends in resistance independent of the pandemic. The near-doubling of annual isolate volume between 2021 and 2023 is itself consistent with changing laboratory activity over the period. The observed trajectory should accordingly be read as a signal warranting prospective investigation with concurrent collection of antimicrobial consumption and infection prevention process indicators, rather than as evidence that post-pandemic health-system disruption caused the resurgence documented here.

### Predictors and risk factors of multidrug resistance

The species-level resistance pattern observed in this study is biologically plausible and consistent with the literature, particularly the greater multidrug resistance burden in Klebsiella spp. than in E. coli. From a clinical perspective, the key implication is that common first-line agents showed poor activity against MDR isolates, whereas colistin and tigecycline retained comparatively better activity, supporting the need for stewardship-guided empiric therapy and strengthened infection prevention measures.

The cooperative nature of beta-lactamase expression, wherein extracellularly secreted beta-lactamases confer phenotypic resistance not only to producing cells but also to sensitive neighboring cells through public goods mechanisms, creates a fitness advantage for Klebsiella strains carrying conjugative plasmids in beta-lactam-rich ICU environments, thereby promoting both plasmid transfer efficiency and the establishment of highly resistant endemic clones (Wang et al., 2023). In contrast, E. coli isolates demonstrated significantly lower odds of multidrug resistance (adjusted odds ratio 0.27; 95% confidence interval 0.19–0.38), suggesting that despite this species’ well-documented global significance as a source of resistance, it exhibits a comparatively reduced propensity for accumulating multiple resistance traits in this particular ICU setting, a finding potentially reflecting differences in plasmid replication systems, conjugation efficiency, or the cost of carrying multiple resistance determinants that may differentially select against polypharmacological E. coli genotypes under intermittent antibiotic pressure (Wang et al., 2023; Shao et al., 2025). Notably, demographic variables, including patient age and biological sex, were not statistically significant predictors of multidrug resistance in the multivariable model, a finding entirely consistent with the global epidemiological literature emphasizing that antimicrobial resistance is fundamentally a microbial and environmental phenomenon rather than a host-factor-driven outcome (Wang et al., 2023; Shao et al., 2025). The association between specimen type and resistance pattern, with sputum samples demonstrating disproportionately high MDR prevalence (44.9%), reflects the microecological reality that ventilated patients undergo protracted exposure to broad-spectrum beta-lactams (particularly carbapenems and third-generation cephalosporins) administered empirically for suspected ventilator-associated pneumonia, thereby creating an intensely selective environment within lower respiratory tract secretions that are preferentially enriched for highly resistant gram-negative bacilli, which is substantially attenuated in urinary or blood specimens collected from patients with less intensive antimicrobial exposure histories (Awad et al., 2017; Martin-Loeches, 2020; Reyes et al., 2023).

Beyond the organism-level predictors identified in the multivariable model, the published literature consistently implicates host comorbidities and prior antimicrobial exposure as critical independent predictors of multidrug-resistant infection in ICU settings, and the absence of these variables from our dataset represents a substantive analytical gap. Diabetes mellitus impairs neutrophil chemotaxis, phagocytic activity, and oxidative burst capacity, and the immune dysregulation it induces promotes prolonged and recurrent infections requiring repeated antimicrobial courses that create cumulative selective pressure for resistance acquisition (Karukappadath et al., 2023; Darwitz et al., 2024). Chronic kidney disease particularly end-stage renal disease requiring hemodialysis independently increases MDR pathogen risk through recurrent vascular access procedures, frequent hospitalization, and repeated antibiotic courses, all of which sustain both colonization pressure and resistance selection (Su et al., 2018; Zilberman-Itskovich et al., 2022). Malignancy and immunosuppressive therapy disrupt mucosal barrier integrity and impair pathogen clearance, necessitating prolonged broad-spectrum therapy and facilitating intestinal colonization with MDR Enterobacterales that frequently precedes invasive infection; colonization rates with carbapenem-resistant and ESBL-producing Enterobacterales of 22% and 19%, respectively, have been documented in patients with hematological malignancies (Luo et al., 2024). Critically, prior antibiotic exposure particularly within the preceding 90 days is one of the most consistently demonstrated independent predictors of MDR isolation in ICU cohorts; prior fluoroquinolone exposure has been identified as the principal modifiable predictor of carbapenem-resistant Enterobacter acquisition (aOR 2.94; p = 0.04), and duration of antibiotic exposure before ICU admission has been independently associated with MDRO acquisition (OR 1.16 per day; 95% CI 1.1–1.2) (Ma et al., 2015; Mendel et al., 2019).

The multivariable model presented here explained 10.5% of the variance in multidrug resistance status (pseudo-R² = 0.105), indicating that the demographic, specimen and organism variables available in the laboratory database account for only a small fraction of the variation observed. Established clinical predictors of multidrug resistance — prior antimicrobial exposure, comorbidity burden, invasive device exposure, length of stay, mechanical ventilation and previous healthcare contact were not recorded in the retrospective dataset and could not be entered into the model. These predictors are consistently associated with multidrug-resistant infection in ICU cohorts elsewhere and are likely to contribute substantially to the unexplained variance, although their relative contribution in this population cannot be estimated from the present data. The model should therefore be regarded as characterizing the microbiological structure of resistance in this cohort rather than as a predictive model of individual patient risk.

Future prospective studies should collect, at minimum: Charlson Comorbidity Index score, diabetes and immunosuppression status, renal replacement therapy dependence, number and class of antibiotics received in the 90 days prior to culture-positive episode, and device-days (urinary catheter, central venous catheter, mechanical ventilation). These variables would substantially improve model explanatory power and enable the development of locally validated clinical risk scores for MDR prediction at ICU admission.

### Antibiotic susceptibility profile and therapeutic implications

The antimicrobial susceptibility landscape revealed across this cohort demonstrated profound limitations in therapeutic options, with severe and widespread resistance to fluoroquinolones, trimethoprim-sulfamethoxazole, and all beta-lactam combinations examined. Ciprofloxacin resistance reached 95.4% among multidrug-resistant isolates, consistent with global patterns wherein Klebsiella spp. accumulate high-level quinolone resistance through a combination of chromosomal mutations in quinolone resistance-determining regions (QRDR) of the gyrA and parC genes and plasmid-mediated quinolone resistance (PMQR) determinants, including qnrA, qnrB, and aac(6’)-Ib-cr genes. Qualitative *in vitro* studies demonstrated that gyrA mutations produced a greater effect on phenotypic resistance than plasmid-mediated mechanisms; however, the co-occurrence of both mechanisms in Klebsiella strains results in highly stable, cross-resistant fluoroquinolone phenotypes refractory to quinolone monotherapy (Tang et al., 2017; Onishi et al., 2022). Resistance to trimethoprim-sulfamethoxazole similarly reflects the plasmid-mediated carriage of dihydrofolate reductase genes (dfrA variants) frequently co-localized on multireplicator plasmids along with carbapenemase determinants, representing a cost-free or low-cost acquisition of additional resistance determinants in an environment where triple-drug-resistant isolates are already maintained by carbapenem pressure (Tang et al., 2017). In contrast, colistin retained near-complete activity against the genera in which it is clinically applicable, with resistance in only 1.2% (9/735 tested) of isolates lacking intrinsic polymyxin resistance. Tigecycline resistance was 17.4% among the 155 isolates in which it was tested, though the low testing coverage of that agent limits confidence in this figure. Together these agents represent a narrow but critical reservoir of therapeutic capacity that must be carefully preserved through urgent antimicrobial stewardship interventions (Moolla et al., 2021; Gómara-Lomero et al., 2022). These agents function as antibiotics of last resort for the treatment of carbapenem-resistant Enterobacterales (CRE) lacking alternative therapeutic options, particularly in resource-limited settings where newer agents with potent activity against resistant gram-negative bacteria remain inaccessible or prohibitively expensive. However, the identification of acquired colistin resistance in 1.2% of isolates lacking intrinsic polymyxin resistance occurring exclusively among multidrug-resistant isolates, in which the proportion was 3.2% represents an early warning signal meriting immediate surveillance attention. Mounting evidence from Africa and globally documents the emergence and geographic dissemination of mobile colistin resistance genes, particularly mcr-1, mcr-3, mcr-8, mcr-9, and mcr-10, which are encoded by plasmids capable of horizontal transfer among Enterobacteriaceae and other gram-negative species (Kigen et al., 2024; Mondal et al., 2024). In Africa, mcr genes have been recovered from human clinical isolates, animal reservoir strains, foods of animal origin, and environmental aquatic and terrestrial ecosystems. They are often co-located on conjugative plasmids with Extended-Spectrum Beta-Lactamase (ESBL) genes or metallo-beta-lactamase (MBL) determinants, creating multidrug-resistant phenotypes resistant to both beta-lactams and polymyxins, rendering such isolates virtually untreatable with available monotherapeutic agents (Anyanwu et al., 2021; Kigen et al., 2024). The presence of acquired colistin resistance in this Somali cohort, confined entirely to multidrug-resistant isolates, coupled with global evidence of escalating tigecycline resistance driven by mobile tet(X) and tmexCD-toprJ genes, underscores the urgent need to implement immediate institutional and national-level interventions to restrict inappropriate empirical colistin use, limit tigecycline exposure to rigorously confirmed cases of untreatable multidrug-resistant or extensively drug-resistant infections, and establish prospective microbiological surveillance for emerging resistance to these final-line agents (Anyanwu et al., 2022). The findings mandate rapid revision of local empirical treatment guidelines and antimicrobial stewardship protocols at tertiary hospitals, emphasizing earlier switching from empirical broad-spectrum regimens to narrower-spectrum agents upon availability of culture results, optimization of dosing regimens for both traditional agents (aminoglycosides, fosfomycin) and last-line therapeutics, and careful consideration of combination therapies wherein colistin or tigecycline are paired with agents such as azithromycin, rifampicin, or fluoroquinolones to maximize bactericidal effects while minimizing the duration of last-line antibiotic exposure (Karaiskos et al., 2019; Gómara-Lomero et al., 2022).

Interpretation of colistin resistance in *Enterobacterales* requires that three mechanistically distinct phenomena be distinguished, since they carry different epidemiological and clinical implications. The first is intrinsic resistance, exhibited by *Proteus* spp., *Providencia* spp., *Morganella morganii* and *Serratia marcescens*, in which the *arnBCADTEF* (*pmrHFIJKLM*) operon is constitutively expressed and lipid A is decorated with 4-amino-4-deoxy-L-arabinose; the resulting reduction in the net anionic charge of the lipopolysaccharide abolishes the electrostatic interaction on which polymyxin binding depends. Intrinsic resistance is a fixed taxonomic property, is not transmissible, and carries no surveillance significance; isolates of these genera must be excluded from colistin resistance denominators, as has been done in the present analysis. The second is chromosomal mutational resistance, arising from mutations in the two-component regulatory systems *pmrAB* and *phoPQ*, or from inactivation of the negative regulator *mgrB* the dominant mechanism in *Klebsiella pneumoniae* each of which de-represses the same lipid A modification pathway. This mechanism is vertically inherited, is frequently selected during colistin therapy, and typically produces high-level, stable resistance. The third is plasmid-mediated resistance conferred by *mcr* genes encoding phosphoethanolamine transferases, which is horizontally transmissible, usually confers lower MICs, and is of the greatest public health concern because of its dissemination potential across species and across the human–animal–environment interface. Superimposed on all three is heteroresistance, in which a resistant subpopulation persists within an otherwise non-resistant isolate at frequencies below the detection threshold of standard broth microdilution; heteroresistance is well documented in *K. pneumoniae* and *Enterobacter* spp., can be selected rapidly during therapy, and means that phenotypic broth microdilution the method applied here systematically under-ascertains the true burden of colistin resistance. The distribution observed in the present cohort is suggestive on this point: all nine colistin-resistant isolates were multidrug-resistant, and six of the nine were *Klebsiella* spp., a pattern more compatible with therapy-selected chromosomal resistance within an already-resistant population than with independent plasmid acquisition, although with only nine isolates this observation is hypothesis-generating rather than conclusive. Because molecular characterization was not available in this retrospective dataset, the resistant isolates cannot be attributed to a specific mechanism, and the reported figure should be read as a phenotypic, conservative lower bound. Determining whether the resistance observed here is *mcr*-mediated, and therefore transmissible, or chromosomally encoded, and therefore clonal, is the single highest-priority question for follow-up work, and should be addressed by targeted PCR for *mcr-1* to *mcr-10*, sequencing of *mgrB*, *pmrAB* and *phoPQ*, and whole-genome sequencing of all colistin-resistant isolates (Anyanwu et al., 2021; Kigen et al., 2024; Mondal et al., 2024).

### Infection control, antimicrobial stewardship, and policy imperatives

The Somali context presents a uniquely urgent and complex challenge for antimicrobial resistance mitigation, as Somalia currently lacks a functional national antimicrobial resistance surveillance system, established WHO GLASS participation framework, or formally adopted the National Action Plan on Antimicrobial Resistance, a surveillance and policy deficit that stands in sharp contrast to its regional peers, where two-thirds of WHO African Region countries have now developed NAPs and 30 of 47 countries participate in WHO GLASS (Fuller et al., 2022; Hassan et al., 2024; Hope et al., 2024). This institutional absence indicates that the resistance data documented in this study represent isolated local intelligence rather than systematically integrated regional epidemiological knowledge capable of informing evidence-based empirical therapy guidelines across multiple healthcare facilities. At the hospital level, immediate actionable interventions must include the establishment of a quarterly institutional antibiogram that systematically catalogs the susceptibility patterns of common nosocomial pathogens stratified by specimen source and patient population, thereby providing contemporary local data to guide empirical therapy protocols and track temporal trends in resistance (Kursheed et al., 2023). The creation of a multidisciplinary Antimicrobial Stewardship Committee composed of infectious disease physicians, clinical microbiologists, pharmacists, infection prevention and control specialists, and hospital administration would enable a systematic review of prescribing practices, identification of inappropriate antimicrobial use, development of pathogen- and site-specific treatment algorithms, and establishment of prospective audit and feedback mechanisms to promote adherence to institutional guidelines (Moolla et al., 2021). Carbapenem and colistin use must be subjected to restricted-use protocols requiring infectious disease consultation and documented clinical justification prior to initiation, with regular audits of appropriateness and educational feedback to prescribers, which have demonstrated efficacy in reducing resistance selection pressure and preserving the susceptibility of key agents in diverse healthcare systems (Abushanab et al., 2022; Chauvin et al., 2025). Furthermore, formal infection prevention and control programs must be strengthened through regular training on hand hygiene, source isolation of patients with multidrug-resistant colonization or infection, environmental cleaning protocols, and surveillance for healthcare-associated infection cluster interventions that address the environmental and behavioral drivers of nosocomial transmission, and reduce the denominator of patients requiring broad-spectrum antimicrobials (Fuller et al., 2022; Kamere et al., 2022). Somalia’s AMR response must be rapidly integrated within the WHO GLASS framework and the WHO Global Action Plan on Antimicrobial Resistance, beginning with national engagement with WHO representatives to establish technical assistance for laboratory quality assurance, standardized antimicrobial susceptibility testing protocols, and data management infrastructure using the WHONET software (Fuller et al., 2022; Hope et al., 2024). The development of a Somalia-specific National Action Plan on Antimicrobial Resistance aligned with WHO recommendations and the One Health approach would create political commitment; secure governmental financing for laboratory and surveillance infrastructure; and establish intersectoral coordination mechanisms linking human health, animal agriculture, and environmental sectors to address the full ecology of resistance amplification (Fuller et al., 2022; Kamere et al., 2022). Without urgent institutional and national-level action, the trajectory evident in this ICU characterized by escalating resistance from 2021 onward will likely persist and diffuse throughout the Somali healthcare system. The trajectory documented in this ICU is consistent with global data reporting 30-day mortality rates of 50–57% among ICU patients with carbapenem-resistant Enterobacteriaceae bacteremia (Baek et al., 2024), underscoring the clinical urgency of the resistance escalation observed.

The establishment of a national sentinel hospital AMR surveillance network, modeled on the WHO GLASS framework and initially anchored at three to five geographically and ecologically representative hospitals, would represent a feasible and cost-effective first step toward generating multicenter data. Such a network would require standardized laboratory quality assurance, unified susceptibility testing protocols (CLSI or EUCAST), and systematic data submission to a central database, enabling the identification of regional resistance gradients, outbreak clusters, and secular trends in resistance that cannot be inferred from single-center experience.

### Limitations

Several limitations should be considered when interpreting these findings. First, the single-center design at a high-acuity national referral hospital introduces selection bias: the complex case-mix and pre-transfer antimicrobial exposure of patients referred from across Somalia likely inflate MDR prevalence relative to community and district-level facilities. Furthermore, Somalia’s regions (Puntland, Somaliland, and the South-Central zones) differ substantially in antimicrobial access, population density, and healthcare infrastructure, meaning the resistance ecology documented in Mogadishu cannot be assumed nationally representative. Multicenter studies incorporating standardized susceptibility testing and WHO WHONET data infrastructure across geographically representative sites are urgently needed to generate nationally valid resistance estimates.

Second, this study relied solely on laboratory records, and clinically important variables including prior antimicrobial exposure, comorbidity indices (e.g. Charlson Comorbidity Index or APACHE II scores), device use (central venous catheters, endotracheal tubes, urinary catheters), surgical history, and immunosuppressive therapy were unavailable. Important clinical risk factors for multidrug resistance, including prior antibiotic exposure, comorbidities, invasive device use, length of hospital stay, mechanical ventilation, dialysis requirement, and previous healthcare contact, could therefore not be incorporated into the regression model, introducing unmeasured confounding. The model’s explanatory power was correspondingly low (pseudo-R² = 0.105), and the magnitude of the contribution made by these unmeasured predictors cannot be determined from these data. Endotracheal aspirates and sterile body fluid specimens were not available as separately analyzable categories in the extracted retrospective dataset and therefore were not reported independently in the present analysis.

Third, respiratory specimens were largely represented by sputum-category samples, while bronchoalveolar lavage and other advanced lower respiratory tract sampling methods were not separately available in the retrospective dataset, which may have limited microbiological characterization of ventilated ICU patients. Because specimen-quality indicators for sputum were not consistently captured in the retrospective database, sputum quality could not be formally evaluated in this analysis, and misclassification of colonization versus true lower respiratory tract infection cannot be excluded.

In addition, colistin categorization relied on phenotypic broth microdilution without molecular confirmation, and heteroresistant subpopulations which are not detected by standard broth microdilution and have been reported in *Klebsiella pneumoniae* and *Enterobacter* spp. may have been misclassified as non-resistant. Genera with intrinsic polymyxin resistance were identified and excluded taxonomically; because species-level confirmation within *Proteus* and *Serratia* was not uniformly available, intrinsic-resistance assignment was made at genus level. Finally, susceptibility testing coverage varied substantially between agents, from 99.7% for colistin to 15.0% for trimethoprim–sulfamethoxazole. Because testing was clinically directed rather than systematic, resistance proportions for agents with low coverage are subject to ascertainment bias in the direction of overestimation, and those estimates should be interpreted as provisional pending systematic panel testing.

The first-episode-per-patient deduplication rule, while avoiding over-representation of patients with repeated cultures, may under-represent Enterobacteriaceae acquired later in an admission following an initial negative or non-Enterobacteriaceae culture.

## Conclusion

This five-year longitudinal surveillance study documents the epidemiology and escalating burden of multidrug-resistant Enterobacteriaceae (MDRE) in the ICU of a tertiary referral hospital in Somalia. The overall MDR prevalence of 37.9%, rising to 54% by 2024, represents an alarming resistance trajectory. Klebsiella spp. was the predominant MDRE organism, with a significantly higher MDR burden than Escherichia coli (adjusted odds ratio 0.27 for E. coli relative to Klebsiella; 95% CI 0.19–0.38). Among tested multidrug-resistant isolates, resistance reached 95.4% for ciprofloxacin, 92.9% for trimethoprim–sulfamethoxazole and 77.5% for meropenem, severely limiting empiric treatment options. Acquired colistin resistance of 1.2% (9/735 isolates from genera without intrinsic polymyxin resistance), occurring exclusively among multidrug-resistant isolates, signals the early erosion of last-resort therapeutic capacity and demands immediate surveillance attention.

As the first longitudinal peer-reviewed AMR study from any Somali ICU, these findings provide an essential baseline for national surveillance efforts. Urgent institutional responses must include the establishment of a quarterly antibiogram, a multidisciplinary Antimicrobial Stewardship Committee, and restricted-use protocols for carbapenems and colistin. At the national level, formal engagement with the WHO GLASS framework and development of a Somalia-specific National Action Plan on Antimicrobial Resistance incorporating multicenter surveillance, molecular resistance characterization, and One Health coordination are essential to prevent further erosion of therapeutic efficacy across Somali healthcare settings. Given the single-center design, the present findings should be interpreted as indicative of high-acuity tertiary ICU settings in urban Somalia; multicenter prospective studies are needed to generate nationally representative resistance data.

Given the single-center design of this study, the reported resistance rates should be interpreted as indicative of the resistance landscape in high-acuity tertiary ICU settings in urban Somalia rather than as nationally representative estimates. Multicenter prospective surveillance studies are essential to validate and contextualize these findings across Somalia’s diverse healthcare settings.

## Data Availability

The raw data supporting the conclusions of this article will be made available by the authors, without undue reservation.

## References

[B1] Abayneh M. Zeynudin A. Tamrat R. Tadesse M. Tamirat A. (2023). Drug resistance and extended-spectrum β-lactamase (ESBLs) - producing Enterobacteriaceae, Acinetobacter and Pseudomonas species from the views of one-health approach in Ethiopia: a systematic review and meta-analysis. One Health Outlook 5, 12. doi: 10.1186/S42522-023-00088-Z 37697359 PMC10496308

[B2] Abushanab D. Nasr Z. G. Al-Badriyeh D. (2022). Efficacy and safety of colistin versus tigecycline for multi-drug-resistant and extensively drug-resistant Gram-negative pathogens—a meta-analysis. Antibiotics 11, 1630. doi: 10.3390/ANTIBIOTICS11111630 36421274 PMC9686723

[B3] Adhikary K. Ganguly K. Chakrabarty S. Sarkar R. Nag R. Srivastava S. . (2025). Understanding antimicrobial resistance: From mechanisms to public health implications. Curr. Pharm. Bio/Technol. 26, 1–16. doi: 10.2174/0113892010406135250828050228 40947740

[B4] Ajulo S. Awosile B. (2024). Global antimicrobial resistance and use surveillance system (GLASS 2022): Investigating the relationship between antimicrobial resistance and antimicrobial consumption data across the participating countries. PloS One 19, e0297921. doi: 10.1371/JOURNAL.PONE.0297921 38315668 PMC10843100

[B5] Alemayehu E. Fiseha T. Gedefie A. Alemayehu Tesfaye N. Ebrahim H. Ebrahim E. . (2023). Prevalence of carbapenemase-producing Enterobacteriaceae from human clinical samples in Ethiopia: a systematic review and meta-analysis. BMC Infect. Dis. 23, 277. doi: 10.1186/S12879-023-08237-5 37138285 PMC10155349

[B6] Anyaegbunam Z. K. G. Mba I. E. Doowuese Y. Anyaegbunam N. J. Mba T. Aina F. A. . (2023). Antimicrobial resistance containment in Africa: Moving beyond surveillance. Biosaf. Health 6, 50. doi: 10.1016/J.BSHEAL.2023.12.003 40078303 PMC11894975

[B7] Anyanwu M. U. Nwobi O. C. Okpala C. O. R. Ezeonu I. M. (2022). Mobile tigecycline resistance: An emerging health catastrophe requiring urgent one health global intervention. Front. Microbiol. 13, 808744. doi: 10.3389/FMICB.2022.808744 35979498 PMC9376449

[B8] Anyanwu M. U. Okpala C. O. R. Chah K. F. Shoyinka V. S. (2021). Prevalence and traits of mobile colistin resistance gene harbouring isolates from different ecosystems in Africa. BioMed. Res. Int. 2021, 6630379. doi: 10.1155/2021/6630379 33553426 PMC7847340

[B9] Awad L. S. Abdallah D. I. Mugharbil A. M. Jisr T. H. Droubi N. S. El-Rajab N. A. . (2017). An antibiotic stewardship exercise in the ICU: building a treatment algorithm for the management of ventilator-associated pneumonia based on local epidemiology and the 2016 Infectious Diseases Society of America/American Thoracic Society guidelines. Infect. Drug Resist. 11, 17. doi: 10.2147/IDR.S145827 29317840 PMC5743123

[B10] Baek M. S. Kim J. H. Park J. H. Kim T. W. Jung H. I. Kwon Y. S. (2024). Comparison of mortality rates in patients with carbapenem-resistant Enterobacterales bacteremia according to carbapenemase production: a multicenter propensity-score matched study. Sci. Rep. 14, 1–9. doi: 10.1038/S41598-023-51118-9 38182719 PMC10770160

[B11] Bitew A. Tsige E. (2020). High prevalence of multidrug-resistant and extended-spectrum β-lactamase-producing Enterobacteriaceae: A cross-sectional study at Arsho Advanced Medical Laboratory, Addis Ababa, Ethiopia. J. Trop. Med. 2020, 6167234. doi: 10.1155/2020/6167234 32411256 PMC7210541

[B12] Cartledge K. Short F. L. Hall A. Lambert K. McDonald M. J. Lithgow T. (2025). Ethical bioprospecting and microbial assessments for sustainable solutions to the AMR crisis. IUBMB Life 77, e2931. doi: 10.1002/IUB.2931 39718471 PMC11668235

[B13] Chauvin C. Jouy E. Chevance A. Jaureguy C. Sauzea X. Jarrige N. . (2025). Decrease in colistin resistance among young bovine and pig productions in France: relationship with colistin usage and stewardship measures. J. Antimicrob. Chemother. 80(12):3227–3235. doi: 10.1093/JAC/DKAF322 41105558

[B14] Darwitz B. P. Genito C. J. Thurlow L. R. (2024). Triple threat: how diabetes results in worsened bacterial infections. Infect. Immun. 92. doi: 10.1128/IAI.00509-23 38526063 PMC11385445

[B15] Fuller W. L. Hamzat O. T. Aboderin A. O. Gahimbare L. Kapona O. Yahaya A. A. . (2022). National action plan on antimicrobial resistance: An evaluation of implementation in the World Health Organization Africa region. J. Public Health Afr. 13, 5. doi: 10.4081/JPHIA.2022.2000 36051526 PMC9425955

[B16] Gahimbare L. Mwamelo A. J. Yahya A. A. Fuller W. Padiyara P. Prakash P. . (2024). Monitoring progress on antimicrobial resistance (AMR) response in the World Health Organization African region: Insights from the Tracking AMR Country Self-Assessment Survey (TrACSS) 2021 results for the human health sector. J. Public Health Afr. 14, 2392. doi: 10.4081/JPHIA.2023.2392 38500695 PMC10946299

[B17] Gómara-Lomero M. López-Calleja A. I. Rezusta A. Aínsa J. A. Ramón-García S. Miral D. . (2022). Repurposing azithromycin in combination with last-line fosfomycin, colistin and tigecycline against multi-drug resistant Klebsiella pneumoniae. BioRxiv, 2022.07.03.498633. doi: 10.1101/2022.07.03.498633 38621210

[B18] Gulumbe B. H. Danlami M. B. Abdulrahim A. (2024). Closing the antimicrobial stewardship gap - a call for LMICs to embrace the global antimicrobial stewardship accreditation scheme. Antimicrob. Resist. Infect. Control 13, 1–3. doi: 10.1186/S13756-024-01371-Y 38355604 PMC10865597

[B19] Hassan S. A. Mohamed Dirie A. Ahmed N. R. Omar A. I. (2024). Update on antimicrobial resistance in Somalia: Current status, challenges, opportunities, and future perspectives. Heliyon 10, e39434. doi: 10.1016/j.heliyon.2024.e39434 39506942 PMC11538744

[B20] Hedberg P. Ternhag A. Giske C. G. Strålin K. Özenci V. Johansson N. . (2022). Ventilator-associated lower respiratory tract bacterial infections in COVID-19 compared with non-COVID-19 patients∗. Crit. Care Med. 50, 825–836. doi: 10.1097/CCM.0000000000005462 35148524 PMC9005099

[B21] Hope M. Kiggundu R. Tabajjwa D. Tumwine C. Lwigale F. Mwanja H. . (2024). Progress on implementing the WHO-GLASS recommendations on priority pathogen-antibiotic sensitivity testing in Africa: A scoping review. Wellcome Open Res. 9, 692. doi: 10.12688/WELLCOMEOPENRES.23133.1 39931110 PMC11809157

[B22] Kamere N. Garwe S. T. Akinwotu O. O. Tuck C. Krockow E. M. Yadav S. . (2022). Scoping review of national antimicrobial stewardship activities in eight African countries and adaptable recommendations. Antibiotics 11. doi: 10.3390/antibiotics11091149 36139929 PMC9495160

[B23] Karaiskos I. Lagou S. Pontikis K. Rapti V. Poulakou G. (2019). The “Old” and the “New” antibiotics for MDR Gram-negative pathogens: For whom, when, and how. Front. Public Health 7. doi: 10.3389/fpubh.2019.00151 31245348 PMC6581067

[B24] Kariuki S. Kering K. Wairimu C. Onsare R. Mbae C. (2022). Antimicrobial resistance rates and surveillance in Sub-Saharan Africa: Where are we now? Infect. Drug Resist. 15, 3589. doi: 10.2147/IDR.S342753 35837538 PMC9273632

[B25] Karukappadath R. M. Sirbu D. Zaky A. (2023). Drug-resistant bacteria in the critically ill: patterns and mechanisms of resistance and potential remedies. Front. Antibiot. 2, 1145190. doi: 10.3389/FRABI.2023.1145190 39816646 PMC11732010

[B26] Kigen C. Muraya A. Wachira J. Musila L. (2024). The first report of the mobile colistin resistance gene, mcr-10.1, in Kenya and a novel mutation in the phoQ gene (S244T) in a colistin-resistant Enterobacter cloacae clinical isolate. Microbiol. Spectr. 12. doi: 10.1128/SPECTRUM.01855-23 38230935 PMC10846102

[B27] Kursheed F. Tabassum A. Farwa U. Wazir S. Shafiq M. Sheikh A. K. (2023). The antibiogram of pus cultures in federal tertiary care hospital, Islamabad and its utility in antimicrobial stewardship. Iran. J. Microbiol. 16, 56–61. doi: 10.18502/IJM.V16I1.14871 38682051 PMC11055436

[B28] Legese M. H. Asrat D. Swedberg G. Hasan B. Mekasha A. Getahun T. . (2022). Sepsis: emerging pathogens and antimicrobial resistance in Ethiopian referral hospitals. Antimicrob. Resist. Infect. Control 11, 83. doi: 10.1186/S13756-022-01122-X 35698179 PMC9195281

[B29] Lord J. Gikonyo A. Miwa A. Odoi A. (2021). Antimicrobial resistance among Enterobacteriaceae, Staphylococcus aureus, and Pseudomonas spp. isolates from clinical specimens from a hospital in Nairobi, Kenya. PeerJ 9. doi: 10.7717/PEERJ.11958 34557345 PMC8418212

[B30] Luo H. Chen X. Jiang Z. Yan Q. (2024). Prevalence of and risk factors for intestinal colonisation by multidrug-resistant Gram-negative bacteria in patients with haematological Malignancies: A systematic review and meta-analysis. Int. J. Antimicrob. Agents 63, 107043. doi: 10.1016/J.IJANTIMICAG.2023.107043 38040318

[B31] Ma X. Wu Y. Li L. Xu Q. Hu B. Ni Y. . (2015). First multicenter study on multidrug resistant bacteria carriage in Chinese ICUs. BMC Infect. Dis. 15, 358. doi: 10.1186/S12879-015-1105-7 26290050 PMC4545921

[B32] Maduko W. Olamijuwon E. Kesby M. Hale J. M. (2025). Public-targeted interventions addressing antimicrobial resistance and antibiotic use in Sub-Saharan Africa: A scoping review. BMJ Glob. Health 10, 17455. doi: 10.1136/BMJGH-2024-017455 40118465 PMC11931924

[B33] Martin-Loeches I. (2020). Current concepts in community and ventilator associated lower respiratory tract infections in ICU patients. Antibiotics 9, 380. doi: 10.3390/ANTIBIOTICS9070380 32635601 PMC7399936

[B34] Mendel L. C. Katz D. Lazarovitch T. Zaidenstein R. Marchaim D. (2019). 496. Carbapenem-resistant Enterobacter: A case-case–control investigation. Open Forum Infect. Dis. 6, S242. doi: 10.1093/OFID/OFZ360.565

[B35] Mondal A. H. Khare K. Saxena P. Debnath P. Mukhopadhyay K. Yadav D. (2024). A review on colistin resistance: An antibiotic of last resort. Microorganisms 12, 772. doi: 10.3390/MICROORGANISMS12040772 38674716 PMC11051878

[B36] Moolla M. S. Whitelaw A. Decloedt E. H. Koegelenberg C. F. N. Parker A. (2021). Opportunities to enhance antibiotic stewardship: colistin use and outcomes in a low-resource setting. JAC-Antimicrob. Resist. 3. doi: 10.1093/JACAMR/DLAB169 34806008 PMC8599735

[B37] Onishi R. Shigemura K. Osawa K. Yang Y. M. Maeda K. Tanimoto H. . (2022). Impact on quinolone resistance of plasmid-mediated quinolone resistance gene and mutations in quinolone resistance-determining regions in extended spectrum beta lactamase-producing Klebsiella pneumoniae isolated from urinary tract infection patients. Pathog. Dis. 80. doi: 10.1093/FEMSPD/FTAC030 35878410

[B38] Reyes L. F. Rodriguez A. Fuentes Y. V. Duque S. García-Gallo E. Bastidas A. . (2023). Risk factors for developing ventilator-associated lower respiratory tract infection in patients with severe COVID-19: a multinational, multicentre study, prospective, observational study. Sci. Rep. 13, 1–15. doi: 10.1038/S41598-023-32265-5 37085552 PMC10119842

[B39] Sajid L. Pir D. Ali M. Arid S. Abbas F. Shaheen S. . (2025). Molecular epidemiology and public health implications of blaCTX-M gene among extended-spectrum beta-lactamase-producing Klebsiella pneumoniae and Escherichia coli isolates in Pakistan: A review. J. Med. Health Sci. Rev. 2. doi: 10.62019/1KJ84D82

[B40] Shao F. Li D. Wang J. Tuo Z. Wang Z. Wei W. . (2025). Beta-lactamase-mediated antibiotic resistance in urinary tract infections: Mechanisms and therapeutic strategies. Urogenital Tract Infection 20, 67–81. doi: 10.14777/UTI.2550012006

[B41] Siachalinga L. Godman B. Mwita J. C. Sefah I. A. Ogunleye O. O. Massele A. . (2023). Current antibiotic use among hospitals in the sub-Saharan Africa region; findings and implications. Infect. Drug Resist. 16, 2179–2190. doi: 10.2147/IDR.S398223 37077250 PMC10108870

[B42] Su G. Xu H. Riggi E. He Z. Lu L. Lindholm B. . (2018). Association of kidney function with infections by multidrug-resistant organisms: An electronic medical record analysis. Sci. Rep. 8, 13372. doi: 10.1038/s41598-018-31612-1 30190585 PMC6127257

[B43] Tang Y. Shen P. Liang W. Jin J. Jiang X. (2017). A putative multi-replicon plasmid co-harboring beta-lactamase genes blaKPC-2, blaCTX-M-14 and blaTEM-1 and trimethoprim resistance gene dfrA25 from a Klebsiella pneumoniae sequence type (ST) 11 strain in China. PloS One 12, e0171339. doi: 10.1371/JOURNAL.PONE.0171339 28152085 PMC5289562

[B44] Tekele S. G. Teklu D. S. Legese M. H. Weldehana D. G. Belete M. A. Tullu K. D. . (2021). Multidrug-resistant and carbapenemase-producing enterobacteriaceae in addis ababa, Ethiopia. BioMed. Res. Int. 2021, 9999638. doi: 10.1155/2021/9999638 34195291 PMC8214486

[B45] Tweldemedhin M. Muthupandian S. Gebremeskel T. K. Mehari K. Abay G. K. Teklu T. G. . (2022). Multidrug resistance from a one health perspective in Ethiopia: A systematic review and meta-analysis of literature (2015–2020). One Health 14, 100390. doi: 10.1016/J.ONEHLT.2022.100390 35686143 PMC9171526

[B46] Wang Q. Wei S. Silva A. F. Madsen J. S. (2023). Cooperative antibiotic resistance facilitates horizontal gene transfer. ISME J. 17, 846. doi: 10.1038/S41396-023-01393-1 36949153 PMC10203111

[B47] Zilberman-Itskovich S. Elukbi Y. Weinberg Sibony R. Shapiro M. Zelnik Yovel D. Strulovici A. . (2022). The epidemiology of multidrug-resistant sepsis among chronic hemodialysis patients. Antibiotics 11, 1255. doi: 10.3390/ANTIBIOTICS11091255 36140034 PMC9495751

