# Supplementary material for: Epidemiology and predictors of multidrug-resistant Enterobacteriaceae in intensive care units of a tertiary hospital in Somalia: a five-year retrospective study (2019–2024)
Source: Front Antibiot. 2026 Sep 3;5:1889216. doi: 10.3389/frabi.2026.1889216 (PMC13581746; doi:10.3389/frabi.2026.1889216)
Supplement: Supplementary file 1 [file Table1.docx]

**Supplementary Table S1. Annual isolate counts and multidrug resistance prevalence, 2019–2024**

| **Year** | **Isolates tested (n)** | **MDR isolates (n)** | **MDR prevalence (%)** | **95% CI** | **Blood** | **Sputum** | **Urine** |
| --- | --- | --- | --- | --- | --- | --- | --- |
| 2019 | 78 | 19 | 24.4 | 16.2–34.9 | 13 | 40 | 25 |
| 2020 | 101 | 25 | 24.8 | 17.4–34.0 | 24 | 61 | 16 |
| 2021 | 70 | 10 | 14.3 | 7.9–24.3 | 21 | 20 | 29 |
| 2022 | 176 | 64 | 36.4 | 29.6–43.7 | 22 | 127 | 27 |
| 2023 | 180 | 86 | 47.8 | 40.6–55.0 | 32 | 100 | 48 |
| 2024 | 153 | 83 | 54.2 | 46.3–61.9 | 11 | 124 | 18 |
| **Total** | **758** | **287** | **37.9** | **34.5–41.4** | **123** | **472** | **163** |

Confidence intervals are Wilson binomial intervals. Cochran–Armitage test for trend across calendar years: χ² = 43.91, df = 1, p = 3.4 × 10⁻¹¹. Pearson chi-square across the six years: χ² = 55.11, df = 5, p = 1.2 × 10⁻¹⁰.
